# Advancing Medical Education: Assessing Technology‐Enhanced Learning Through the Lens of the Canadian Medical Education Directions for Specialists (CanMEDS) Framework—A Perspective Study

**DOI:** 10.1002/hsr2.71171

**Published:** 2025-08-18

**Authors:** Mahboubeh Asadi, Zahra Karimian

**Affiliations:** ^1^ Student Research Committee Shiraz University of Medical Sciences Shiraz Iran; ^2^ Department of E‐Learning in Medical Sciences, Virtual School and Center of Excellence in E‐Learning Shiraz University of Medical Sciences Shiraz Iran

**Keywords:** CanMEDS model, medical education, technology‐enhanced learning

## Abstract

**Background and Aims:**

The CanMEDS framework is a widely adopted competency‐based model that defines essential roles for physicians to ensure high‐quality patient care. Technology‐enhanced learning (TEL) has emerged as a transformative approach in medical education, offering flexible, interactive, and personalized learning experiences. This study explores the integration of TEL within the CanMEDS framework assessment model to enhance competency evaluation in medical education.

**Methods:**

This perspective study reviews the evolving role of TEL in medical education and its alignment with the seven CanMEDS roles: Medical Expert, Communicator, Collaborator, Leader, Health Advocate, Scholar, and Professional. It examines the benefits and challenges of TEL integration, including innovative assessment methods such as virtual simulations, artificial intelligence, and mobile applications.

**Results:**

TEL provides significant advantages in accessibility, scalability, and cost‐effectiveness of competency assessments. Virtual reality and AI‐driven tools enable realistic clinical scenarios and personalized feedback, improving the evaluation of complex skills. TEL also supports continuous learning and collaboration through digital platforms. However, challenges include ensuring assessment validity and reliability, addressing institutional barriers, faculty training needs, ethical concerns regarding data privacy, and disparities in technology access.

**Conclusion:**

Integrating TEL into the CanMEDS framework enhances medical education by offering innovative, flexible, and effective assessment methods. To maximize benefits, institutions must address technological, ethical, and pedagogical challenges through comprehensive implementation plans, faculty development, and clear guidelines. Emerging technologies hold promise for further advancing competency‐based education, ensuring future physicians are well‐equipped to meet evolving healthcare demands.

## 1 Introduction

When evaluating medical trainees, certain assumptions underlie the process. Effective frameworks should enable educators to determine trainees' readiness for advancement, in other words, whether they have attained the desired level of competence [1]. One such framework that has gained widespread recognition is the Canadian Medical Education Directions for Specialists, also known as CanMEDS. The CanMEDS physician competency framework was developed for the first time in the 1990s and was officially endorsed by the Royal College in 1996. It has since evolved until 2017 [2]. The CanMEDS framework is now used to guide medical education development in numerous countries for both postgraduate and undergraduate education. Frameworks serve as a point of reference for everyone involved in the curriculum and should fulfill the educational purposes and experiences [3].

The CanMEDS framework assessment model serves as a comprehensive guide for assessing the competencies required for physicians to provide high‐quality patient care, and is the basis for the educational and practice standards set by the Royal College of Physicians and Surgeons of Canada [4].

While the CanMEDS framework has been widely adopted, it is important to note that it is not without criticism. Some argue that the framework may oversimplify the complex nature of medical practice and that its implementation can be challenging in diverse healthcare systems. Additionally, the framework has undergone revisions since its inception, with the most recent update in 2015 replacing the “Manager” role with “Leader” [3, 5].

**Figure 1:**
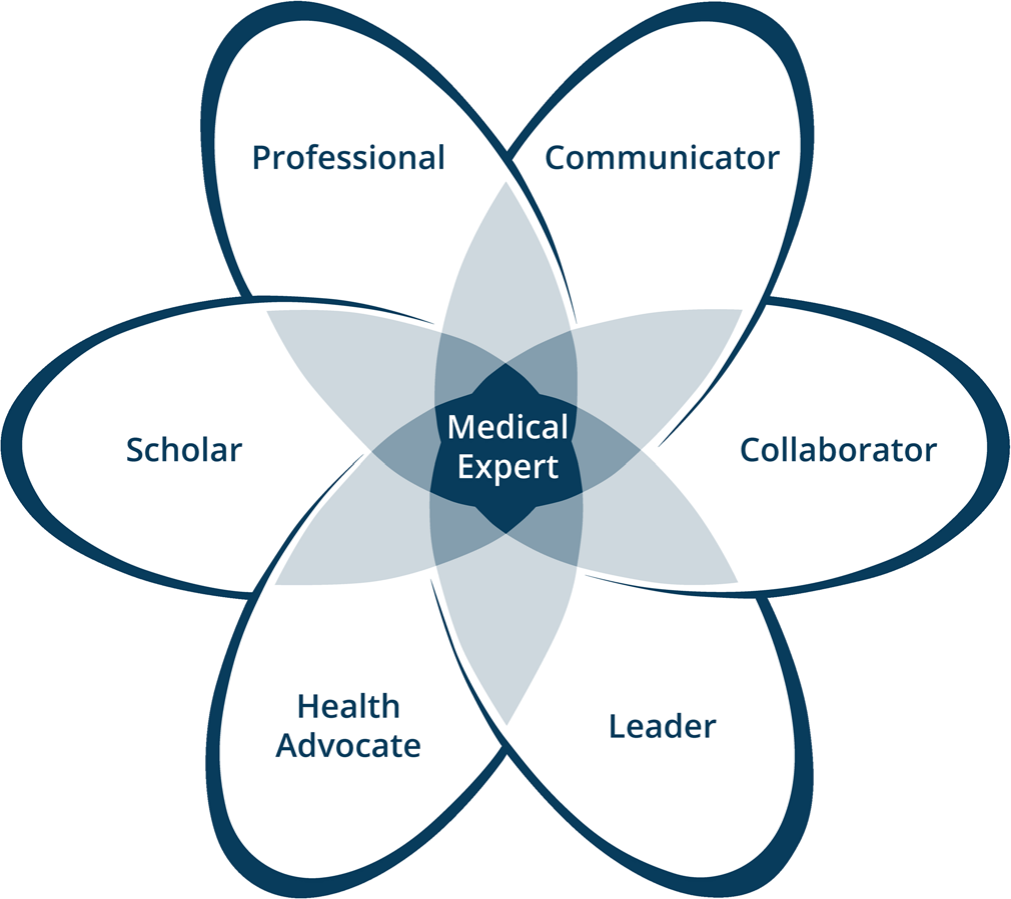
Dimensions of the CanMEDS model [3, 5].

The framework consists of seven roles that physicians must fulfill (Figure 1):

1. Medical Expert: Physicians integrate all of CanMEDS Roles, applying medical knowledge, clinical skills, and professional attitudes in their provision of patient‐centered care. A medical expert is the central physician role in the CanMEDS framework.
2. Communicator: Physicians effectively facilitate the doctor–patient relationship and the dynamic exchanges that occur before, during, and after the medical encounter.
3. Collaborator: Physicians effectively work within a healthcare team to achieve optimal patient care.
4. Leader: Physicians are integral participants in healthcare organizations, organizing sustainable practices, making decisions about resource allocation, and contributing to the effectiveness of the healthcare system by demonstrating collaborative leadership.
5. Health Advocate: Physicians responsibly use their expertise and influence to advance the health and well‐being of individual patients, communities, and populations.
6. Scholar: Physicians demonstrate a lifelong commitment to reflective learning, as well as the creation, dissemination, application, and translation of medical knowledge.
7. Professional: Physicians are committed to the health and well‐being of individuals and society through ethical practice, profession‐led regulation, and high personal standards of behavior.

Each of these descriptors is defined on a more detailed level, as these domains of competence are considered too general for teaching and assessment purposes [5, 6]. In many cases, a further level of detail is added. The CanMEDS framework has 7 roles, 134 “elements,” 28 “key competencies,” and 125 “enabling competencies” [3, 5].

Additionally, after 2015, attention to patient safety was specifically emphasized [2]. This perspective may largely be strengthened by the emergence of new technologies. The existence of innovative technologies such as physical and electronic simulators, virtual reality, and augmented reality provides opportunities to utilize these technologies to enhance patient safety in both treatment and the education of students. It is crucial to consider these evolving perspectives when discussing the integration of technology‐enhanced learning (TEL) within the CanMEDS model.

## 2 Role of the Learning Environment

The learning environment has a major influence on students' satisfaction with their learning experience and is considered a crucial factor in determining their success and quality of life [3]. The impact of the learning environment on students' competency and identity formation in competency‐based clinical education has been well documented. The cultural climate of the learning environment provides a medium for students to interact in a community of practice and develop competency and self‐efficacy. Recently, the learning environment has been proven to be a significant factor in ensuring student well‐being and preventing burnout [7]. However, it is important to recognize that the learning environment is not uniform across institutions and can be influenced by factors such as resource availability, institutional culture, and geographical location. These disparities may be further exacerbated by the introduction of technology‐enhanced learning, potentially widening the gap between well‐resourced and under‐resourced educational settings.

The relationship between the educational environment and achievement, satisfaction, and success of students in medical schools has highlighted the need to the technology‐enhanced learning environment [8]. It seems, the effects of TEL on the CanMEDS framework will be significant.

## 3 Technology‐Enhanced Learning Environment

Medical students often encounter difficulties with complex and extensive knowledge, visualizing body structures, limited access to learning materials, and lack of clinical relevance. To overcome these, innovative approaches must be incorporated into their learning [7].

TEL has revolutionized the field of medical education by providing innovative methods to deliver and assess knowledge and skills [8].

TEL in medical education provides flexibility in time and location for learners and educators, allowing access to resources conveniently and enabling efficient personalized feedback. Technology tailors learning experiences to each student's unique needs, ensuring necessary support for their learning style [9]. TEL provides an interactive educational experience with virtual simulations and augmented reality for safe clinical skills practice, boosting confidence, and enabling immediate feedback [7]. Critics argue that TEL may reduce educator–learner interactions, but this can be addressed with blended learning using online and traditional methods [10].

## 4 Integration of TEL Into the CanMEDS Framework Assessment Model

Traditional assessment methods like written exams or clinical evaluations may not fully capture all competencies. Workplace assessments are challenged by assessor expertise differences and the varied nature of tasks, as well as constant distractions and changes [3]. There is great potential for TEL to improve assessment in medical education within the CanMEDS framework. Using technology for assessment has many benefits, like accessibility, scalability, and cost‐effectiveness. It enables simultaneous assessment of many learners without compromising quality, and often results in lower costs than traditional methods [11, 12].

However, it is crucial to note that the cost‐effectiveness of TEL is not universal and can vary significantly depending on the specific tools and technologies employed. While some TEL solutions may reduce costs in the long term, the initial investment in developing and implementing these technologies can be substantial. Institutions must carefully consider the financial implications and conduct thorough cost–benefit analyses before adopting TEL solutions.

These results illustrate the successful integration of TEL into clinical skills assessment, which can be aligned with the CanMEDS framework. Virtual simulations assess clinical skills effectively by offering realistic scenarios for decision‐making based on patient data and prompt feedback. For instance, OSCE 3D uses 3D imaging and virtual reality to simulate a clinical skills lab, evaluating skills [13]. Emerging tech like AI, VR, and mobile apps could change how competency assessments are done. For instance, AI can analyze student data and give personalized feedback. Simulations can assess complex clinical skills with real‐life scenarios [14]. Mobile applications can provide ongoing formative assessments to promote continuous learning throughout medical training [15]. Therefore, TEL improves assessment of competencies in CanMEDS, leading to better learning experiences and more effective assessment. The outlook of the future world indicates that the emergence of new technologies fundamentally impacts each of the components of the CanMEDS framework.

- For medical experts, Telemedicine enables remote consultations, improving access to care, and AI diagnostics assist in diagnosing conditions through data analysis, enhancing decision‐making. In communicator components—for example, digital communication tools facilitate better patient–provider communication through secure messaging and telehealth platforms. Also, patient portals allow patients to access their health information, enhancing engagement.
- In the collaborator dimension, interdisciplinary platforms and tools like shared electronic health records (EHRs) promote collaboration among healthcare teams. On the other hand, virtual collaboration tools enable real‐time communication and coordination among team members.
- Health system leadership will also benefit from technologies. Artificial intelligence and data analytics are fundamental changes in decision‐making; leaders can use analytics to make informed decisions about resource allocation and operational improvements. Additionally, project management software enhances the organization and tracking of healthcare initiatives.
- In the health advocate component, the development of health apps empowers patients to manage their health proactively, while social media can be used to raise awareness about public health issues and advocate for community health needs.
- The scholar role will be significantly influenced by the development of the digital space and new technologies. Although the extent of these impacts is vast, for example, e‐learning platforms provide access to continuous medical education and training anytime, anywhere, for everyone. Additionally, research databases facilitate the dissemination and application of medical knowledge through online journals and databases.
- Lastly, in the professional component, we can point to the impacts of technology on ethical and professional dimensions. Ethics training software offers simulations and scenarios to enhance understanding of ethical practices. Furthermore, regulatory compliance tools help ensure adherence to professional standards and regulations.

## 5 Challenges of Integration of TEL Into the CanMEDS Framework Assessment Model

TEL provides valuable tools and flexibility for assessing CanMEDS competencies but also has limitations related to validity, reliability, and ethical consideration. Some of these challenges include the following aspects.

### 5.1 Nature and the Methods of Evaluation

Evaluating competencies within the CanMEDS framework requires a comprehensive approach that combines formative and summative assessments. Formative assessments (e.g., quizzes, reflective journals) provide ongoing feedback, support self‐directed learning, and align with roles such as Scholar and Professional. Technology‐enhanced learning enhances these assessments through online platforms and adaptive tools that personalize learning and identify gaps [16]. Summative assessments, such as Objective Structured Clinical Examinations (OSCEs), Directly Observed Practical Skills (DOPS), board exams, and others, serve as high‐stakes evaluations essential for certification and licensure. They assess a broad range of competencies, including clinical skills, communication, and leadership [17, 18, 19]. TEL introduces innovations like virtual simulations and AI‐driven scenarios to assess complex clinical skills remotely and at scale. However, it should also be noted that although TEL offers substantial benefits, its integration into competency‐based assessment models is not without risks. For instance, validity and reliability remain primary concerns, particularly in high‐stakes contexts. The authenticity of virtual simulations and the consistency of automated scoring systems must be rigorously validated to ensure fair and accurate measurement of competencies. Additionally, there is a risk that overreliance on technology may inadvertently narrow the scope of assessment, potentially neglecting essential interpersonal and professional skills that are best observed in authentic clinical environments [19, 20, 21].

### 5.2 Faculty Readiness and Development

The successful integration of TEL into the CanMEDS framework depends largely on the preparedness and adaptability of educators. Faculty members must not only update their teaching and assessment strategies to incorporate new digital tools but also maintain the essential humanistic elements of medical education. Many instructors may initially lack the technological proficiency or pedagogical skills required for effective TEL implementation. Addressing this gap calls for comprehensive and ongoing faculty development initiatives that build both technical competence and educational best practices [19, 22].

### 5.3 Institutional Infrastructure

The development and effective implementation of the CanMEDS framework require strong institutional commitment on multiple fronts. First, dedicated investment in upgrading technological infrastructure—such as high‐speed internet, secure digital platforms, and modern devices—is essential to create an environment conducive to technology‐enhanced learning [23]. Equally important is the provision of comprehensive technical support and ongoing maintenance to ensure smooth operation and prompt resolution of technical issues [23, 24]. Institutional leadership must also prioritize faculty development by offering targeted training programs that build both technological proficiency and pedagogical expertise, thereby empowering educators to effectively utilize TEL tools while maintaining the core values of medical education. Additionally, fostering a supportive organizational culture that values innovation, collaboration, and continuous improvement is crucial. This includes clear communication about the benefits and goals of TEL, recognition of faculty efforts in adopting new methods, and the establishment of feedback mechanisms to address concerns and share best practices. Ultimately, a strategic and holistic approach to strengthening institutional infrastructure not only facilitates the effective integration of TEL but also ensures that technological advancements translate into meaningful improvements in competency‐based medical education [21, 24].

### 5.4 Equity and Access in TEL‐Enhanced Medical Education

The integration of technology‐enhanced learning (TEL) into medical education holds great promise for improving accessibility and personalization of learning experiences. However, it also risks exacerbating existing inequities due to the persistent digital divide. Learners in resource‐limited settings often face significant barriers such as unreliable internet connectivity, lack of access to modern devices, and insufficient institutional support for TEL platforms [24]. These disparities can create uneven learning opportunities, negatively impacting both the educational experience and assessment outcomes. As a result, the fundamental goal of fair and inclusive medical education may be compromised if these challenges are not proactively addressed. Moreover, the variability in technological infrastructure across institutions and geographic regions can widen the gap between well‐resourced and under‐resourced learners, potentially limiting the effectiveness of TEL in achieving the competencies outlined in the CanMEDS framework [25].

### 5.5 Ethical Considerations in TEL‐Enhanced Assessment

The integration of technology‐enhanced learning into medical education assessment introduces complex ethical challenges, particularly regarding the collection, storage, and analysis of learner data. As TEL platforms increasingly rely on digital tools to track student performance, engagement, and progress, large volumes of sensitive personal and academic information are generated and processed. This raises significant concerns about privacy, as unauthorized access or misuse of data could compromise learner confidentiality and trust. Ensuring informed consent is equally vital; students must be clearly informed about what data is collected, how it will be used, and who will have access, empowering them to make knowledgeable decisions about their participation [26].

Data security is another critical issue, as breaches or inadequate protection measures can expose sensitive information to cyber threats or unauthorized parties. Institutions must implement robust technical safeguards, such as encryption and secure authentication protocols, to protect data integrity and confidentiality. Furthermore, clear, transparent, and regularly updated institutional policies are essential for governing data management practices, delineating responsibilities, and outlining procedures for addressing potential breaches or ethical dilemmas [25, 26].

Beyond technical measures, fostering a culture of ethical awareness among faculty and staff is crucial. Educators and administrators should receive training on ethical data handling, privacy laws, and the responsible use of learner information in assessment and feedback. Regular audits and ethical reviews can help ensure ongoing compliance with best practices and legal requirements.

## 6 Conclusion

TEL has made significant improvements to the CanMEDS framework assessment model in medical education by introducing innovative methods for evaluating competency across various dimensions. These methods have brought about increased accessibility, scalability, and cost‐effectiveness. However, there are still concerns regarding the validity and reliability of the assessments, as well as learner engagement and motivation.

Looking toward the future, emerging technologies such as artificial intelligence, simulations, and mobile applications have the potential to enhance assessment within the CanMEDS framework, but they also present new challenges that need to be addressed. To successfully integrate TEL into the CanMEDS framework, we recommend the following strategies:

1. Develop a comprehensive implementation plan that addresses institutional barriers, resource allocation, and faculty development.
2. Establish clear ethical guidelines for the use of technology in assessment, particularly concerning data privacy and security.
3. Conduct pilot studies to evaluate the effectiveness and feasibility of TEL solutions before full‐scale implementation.
4. Foster collaboration between medical educators, technology experts, and ethicists to ensure a holistic approach to TEL integration.
5. Regularly review and update TEL strategies to align with evolving CanMEDS competencies and technological advancements.

By integrating technology as an essential component of medical education while addressing these challenges, we can be confident that future physicians will possess the necessary competencies to deliver high‐quality patient care effectively.

## Author Contributions

**Mahboubeh Asadi:** conceptualization, writing – original draft, data curation, investigation. **Zahra Karimian:** conceptualization, methodology, validation, data curation, supervision, resources, project administration, writing – review and editing. All authors have read and approved the final version of the manuscript.

## Ethics Statement

The authors have nothing to report.

## Consent

The authors have nothing to report.

## Conflicts of Interest

The authors declare no conflicts of interest.

## Transparency Statement

The lead and corresponding author Zahra Karimian affirms that this manuscript is an honest, accurate, and transparent account of the study being reported; that no important aspects of the study have been omitted; and that any discrepancies from the study as planned (and, if relevant, registered) have been explained.

## Data Availability

The authors confirm that the data supporting the findings of this study are available within the article [and/or] its Supporting Materials. The data that support the findings of this study are available from the corresponding author upon reasonable request. Corresponding author [Zahra Karimian] had full access to all of the data in this study and takes complete responsibility for the integrity of the data and the accuracy of the data analysis.
